# The stress-responsive protein REDD1 and its pathophysiological functions

**DOI:** 10.1038/s12276-023-01056-3

**Published:** 2023-09-01

**Authors:** Ji-Yoon Kim, Young-Guen Kwon, Young-Myeong Kim

**Affiliations:** 1 Department of Anesthesiology and Pain Medicine, Hanyang University Hospital, Seoul, 04763 Republic of Korea; 2 Department of Biochemistry, College of Life Science and Biotechnology, Yonsei University, Seoul, 03722 Republic of Korea; 3 Department of Molecular and Cellular Biochemistry, School of Medicine, Kangwon National University, Chuncheon, 24341 Republic of Korea

**Keywords:** Mechanisms of disease, Cell biology

## Abstract

Regulated in development and DNA damage-response 1 (REDD1) is a stress-induced protein that controls various cellular functions, including metabolism, oxidative stress, autophagy, and cell fate, and contributes to the pathogenesis of metabolic and inflammatory disorders, neurodegeneration, and cancer. REDD1 usually exerts deleterious effects, including tumorigenesis, metabolic inflammation, neurodegeneration, and muscle dystrophy; however, it also exhibits protective functions by regulating multiple intrinsic cell activities through either an mTORC1-dependent or -independent mechanism. REDD1 typically regulates mTORC1 signaling, NF-κB activation, and cellular pro-oxidant or antioxidant activity by interacting with 14-3-3 proteins, IκBα, and thioredoxin-interacting protein or 75 kDa glucose-regulated protein, respectively. The diverse functions of REDD1 depend on cell type, cellular context, interaction partners, and cellular localization (e.g., mitochondria, endomembrane, or cytosol). Therefore, comprehensively understanding the molecular mechanisms and biological roles of REDD1 under pathophysiological conditions is of utmost importance. In this review, based on the published literature, we highlight and discuss the molecular mechanisms underlying the REDD1 expression and its actions, biological functions, and pathophysiological roles.

## Cell stress: Damaging activities of a stress-response protein

The gene that codes for the REDD1 protein is activated by a variety of cellular stresses, including metabolic imbalance and DNA damage; REDD1’s effect on various aspects of cellular activities contributes to the pathogenesis of many diseases. Researchers in South Korea led by Young-Myeong Kim at Kangwon National University, Chuncheon, review the cellular functions, molecular mechanisms, and disease-causing actions of REDD1. They assess the extensive evidence on the mechanisms by which REDD1 acts as a detrimental factor in serious conditions, including metabolic disorders, cancer, muscle atrophy, neurological diseases, and autoimmune diseases. However, some of the evidence is uncertain and controversial, and the involvement of REDD1 in disease may depend on complex interactions with other factors, meaning further research is needed to improve understanding. Drugs that regulate the activity of the REDD1 protein or its gene could have therapeutic potential.

## Introduction

Cells and organisms are constantly challenged by changes to environmental and physiological conditions and are exposed to various stressful conditions or stressors, including temperature changes, oxidative stress, low oxygen tension, imbalanced osmolality, and an altered nutrient supply. To maximize cell function and survival under these stress conditions, adaptive pathways are activated through the induction of stress-responsive proteins, called stress proteins. The conserved stress-responsive protein regulated in development and DNA damage 1 (REDD1) is induced by various cellular stressors, including DNA damage, hypoxia, and nutrient imbalance, and plays an important role in the regulation of cellular function and metabolism and in the pathogenesis of various diseases.

Increasing evidence from several independent studies suggests that REDD1 regulates various cellular and metabolic processes, including mitochondrial biogenesis and function^1^, reactive oxygen species (ROS) generation^2^, autophagy^2,3^, protein and lipid synthesis^4–7^, and glycolysis^8^. Increased REDD1 expression is involved in the pathogenesis of several metabolic disorders, including obesity and type 2 diabetes (T2D)^6,7,9^, macular degeneration, diabetic retinopathy^10,11^, muscle atrophy^4,5^, and hepatic steatosis^6,7^, as well as other pathogenic processes, such as neurodegeneration^12,13^, emphysema^14^, tumorigenesis^15–17^, and tumor angiogenesis^8,18^. Therefore, it is conceivable that REDD1 functions as an important regulator of cellular metabolic capacity and contributes to the development of various diseases, although the precise underlying mechanisms have not been elucidated. Considering these possibilities, we review and discuss the cellular functions, molecular mechanisms, and pathophysiological roles of REDD1 under physiological and pathological stress conditions.

### Regulatory effects of cellular stresses on REDD1 expression

REDD1 (also known as RTP801, DDIT4, or Dig2) is encoded by DNA damage-inducible transcript 4 (*DDIT4*), located on human chromosome 10q24.33. This gene was initially found to be expressed in response to cellular stresses in 2002 by two independent groups. Shoshani et al. identified hypoxia-regulated genes in rat C6 glioma cells and cloned *RTP801* as a novel hypoxia-inducible factor (HIF-1)-responsive gene^19^. Ellisen et al. also cloned the same gene but named it *Redd1*, and they found that its expression was induced by DNA damage and during embryogenesis depending on p53 or p63 (a member of the p53 family)^20^. One year later, Wang et al. discovered dexamethasone (DEX)-induced gene 2 (*Dig2*), a homolog of *Redd1*/*RTP801*, in murine T-cell lines and confirmed its upregulation in mouse thymocytes treated with DEX^21^. In 2008, Knowles et al. found that DDIT4 protein expression was upregulated in apoptotic tumor cells induced by the inhibition of fatty acid synthase through either knockdown with small interfering RNA or treatment with the small-molecule drug orlistat^22^. These results suggest that REDD1 is a stress-responsive protein induced by hypoxia, toxic cellular agents, and hormones.

**Fig. 1:**
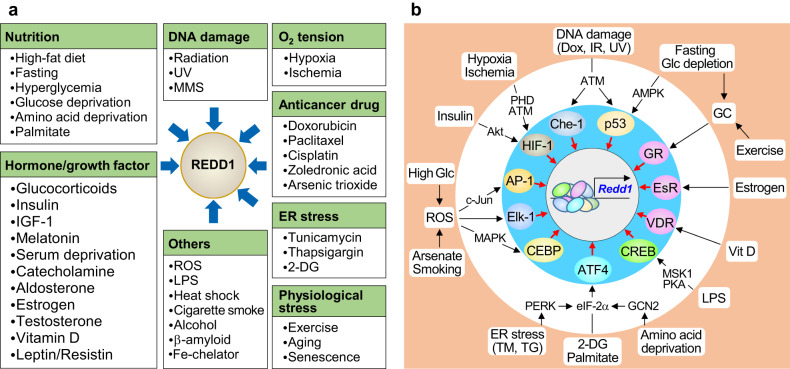
Multiple stresses and transcription factors induce REDD1 expression. **a** Various stresses that stimulate REDD1 expression. 2-DG 2-deoxyglucose, IGF-1 insulin-like growth factor-1, LPS lipopolysaccharide, MMS methyl methane sulfonate, ROS reactive oxygen species, UV ultraviolet. **b** Transcription factors that induce REDD1 expression. Dox doxorubicin, EsR estrogen receptor, GCN2 general control nonderepressible 2, Glc glucose, GR glucocorticoid receptor, IR irradiation, PHD prolyl hydroxylase domain protein, TG thapsigargin, TM tunicamycin, VDR vitamin D receptor.

After *Redd1* cloning, several studies confirmed that this gene could be rapidly induced by multiple stressors (Fig. 1a). Similar to its expression in response to previously identified cellular stressors, for example, hypoxia^15,19^ and ischemia^23,24^, DNA-damaging agents^20^, and ROS^24^, REDD1 was upregulated by metabolic imbalances caused by excessive or deficient nutrients (e.g., high glucose levels, amino acid depletion, high free fatty acid levels, and fasting or starvation)^25–34^. Furthermore, it functions as an adaptive regulator under cellular stress conditions, including as a fat storage modulator^6,9^, anabolic inhibitor^5,27^, or cytotoxic regulator^10,35,36^. REDD1 is also highly expressed in metabolic stress-related diseases, including obesity, T2D, and diabetic retinopathy^6,28,36^.

As a stress hormone-response gene^21^, REDD1 is upregulated by several hormones or growth factors, including glucocorticoids (cortisol, corticosterone, and DEX)^12,22,33,37^, insulin^38,39^, insulin-like growth factor-1^40^, catecholamines (adrenaline and noradrenaline)^41^, aldosterone^42^, estrogen^43^, growth hormones (particularly growth hormone deficiency, such as when serum levels are low)^34^, vitamin D^44,45^, and melatonin^46^. Hormone-responsive REDD1 may induce muscle atrophy by inhibiting mammalian target of rapamycin (mTOR) function, particularly mTOR complex 1 (mTORC1)-mediated protein synthesis^4,5^. In contrast, testosterone suppresses DEX-induced REDD1 expression by binding to the androgen receptor and thus prevents glucocorticoid-mediated suppression of mTORC1 signaling in skeletal muscles^47^.

REDD1 is potentially induced in cultured cells and animal models treated with or exposed to anticancer drugs^18,48–50^, endoplasmic reticulum (ER) stressors^25,51^, cigarette smoke^14^, alcohol^52^, heat shock^21^, β-amyloid (Aβ)^53^, and iron chelators (deferasirox and deferoxamine)^54,55^, likely contributing to the regulation of cell death and autophagy. REDD1 levels are also increased by physiological stress associated with exercise, aging, or senescence^42,55–59^ and may contribute to muscle atrophy^4,58^ or the senescence-associated secretory phenotype^59^. Moreover, REDD1 is upregulated in response to lipopolysaccharide (LPS) and augments inflammatory responses^60–62^. REDD1 is, therefore, considered a stress-responsive protein that regulates metabolism, inflammation, oxidative stress responses, cell and organ homeostasis, and cell death and survival.

### Transcriptional regulation of REDD1

Transcription factors, including HIF-1, p53, and glucocorticoid receptor, have been shown to stimulate REDD1 expression^19–21^, and several studies have shown that some other transcription factors are also involved in REDD1 expression by binding the promoter of its encoding gene^14,48,50^. Figure 1b shows the possible involvement of several transcription factors and related upstream signals in the expression of REDD1 in response to various stressors. Notably, binding sites for the transcription factors HIF-1α, p53, Elk-1, CEBP, hepatic nuclear factor-4, and NF-κB are located in the human REDD1 promoter^50^. HIF-1α activation is mediated by prolyl hydroxylase domain proteins and ataxia-telangiectasia-mutated protein kinase (ATM) under hypoxic and ischemic conditions or via insulin-mediated Akt activation^15,38,63,64^. DNA damage-response transcription factors, including p53 and Che-1, are regulated through ATM activation by chemotherapeutic drugs, hypoxia, and glucose depletion^20,48,64–66^. Fasting or restricted glucose intake increases the cellular AMP/ATP ratio and activates AMPK, which induces p53 stabilization via the phosphorylation of Ser18 in mice (Ser15 in humans)^67,68^, eventually leading to REDD1 upregulation^32^.

The activation of the stress-induced transcription factor ATF4 by the PERK–eIF-2α axis under ER stress or amino acid deprivation conditions is sufficient to induce REDD1 expression^25,29,51^. Additionally, ROS-generating stimulants, including cigarette smoke, arsenate, and hyperglycemia, stimulate REDD1 expression by activating c-Jun/AP-1, CEBP, and p38MAPK/Elk-1^25,50,69–71^. Starvation/fasting or exercise elevates endogenous glucocorticoid levels to stimulate gluconeogenesis in the liver and promotes REDD1 expression through the activation of its nuclear receptor^21,33,72^. Other hormones, including estrogen and vitamin D, also induce REDD1 expression via their specific nuclear receptors^44,45^. Furthermore, the immune activator LPS stimulates REDD1 expression by activating CREB via the early p38MAPK–MSK1 pathway and the late COX-2/PGE2-mediated cAMP–PKA pathway but not via NF-κB activation^60^, even though the NF-κB-binding motif is present in the REDD1 promoter^50^. Thus, REDD1 expression can be regulated by multiple transcription factors in response to various cellular stresses.

### Biochemical and structural properties of REDD1

REDD is ubiquitously expressed at low levels^19^. It consists of 232 amino acids with a predicted molecular weight of 25 kDa and is an acidic serine-rich protein with an evolutionarily conserved sequence at the C-terminus^20^. REDD1 localizes mostly to the cytoplasm^20^ but is also found in the nucleus^50,73^, cellular membranes^15,73^, and mitochondria^24^. REDD1 exhibits multiple biological functions, but its functional motifs or domains have not been clearly identified via amino acid sequence analysis^19,74^; furthermore, no REDD1 enzyme activity has been documented thus far. Vega-Rubin-de-Celis et al. crystallized a portion of human REDD1 comprising amino acids 89–226 with a deletion of the internal hydrophobic sequence ^200^FLPGF^204^ and reported its crystal structure containing two α-helices and four β-sheets^74^. The amino acid sequence ^218^KKKLYSSE^225^ in the C-terminal strand β4 is presumed to be a functionally significant residue that regulates mTORC1 activity^74^. Among these amino acids, Lys219 and Lys220 have been confirmed to play a crucial role in atypical NF-κB activation^6^. Furthermore, the three contiguous lysine residues ^218^KKK^220^ are highly conserved and assumed to be necessary for REDD1 localization to mitochondria^15^ and the plasma or cellular membranes^73^.

Since REDD1 has a short half-life of 5–20 min, its protein level is low in most cells^20,75^. REDD1 levels are increased by the proteasome inhibitor MG-132, indicating that protein stability is regulated via proteasomal degradation^76^. Another independent study showed that REDD1 is phosphorylated at Ser19, Thr23, and Thr25 through the activity of GSK3β and then rapidly degraded by the ubiquitin-mediated degradation system mediated by the CUL4A–DDB1–ROC1–β-TRCP E3 ligase complex^77^. Although REDD1 expression is induced by a variety of cellular stressors, it can also be transcriptionally downregulated through a negative feedback mechanism involving the dysregulation of the mitochondrial ROS−HIF-1α axis^17^. Overall, the biological activity of REDD1 is modulated by transcriptional regulation, proteasomal degradation, and a negative feedback loop.

## Biological functions of REDD1

### REDD1 as a negative regulator of mTORC1

**Fig. 2:**
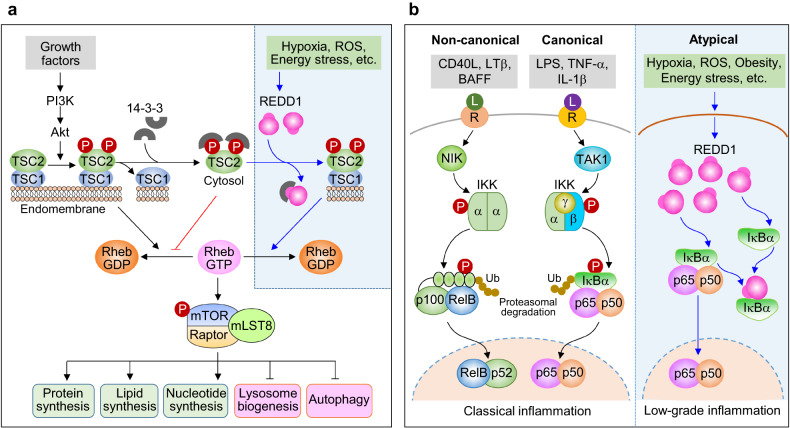
Models of REDD1-mediated mTORC1 inhibition and atypical NF-κB activation. **a** Schematic showing REDD1-mediated mTORC1 inhibition. Growth factor-activated Akt phosphorylates Ser939 and Ser981 in TSC2 (a GTPase-activating protein, GAP) in the endomembrane-bound TSC1/2 complex (active form), facilitates TSC2/14-3-3 association (inactive GAP) in the cytosol, and inhibits GTP hydrolysis of Rheb, resulting in elevated Rheb-GTP levels and mTORC1 activation. Stress-induced REDD1 sequesters 14-3-3, maintains the active TSC1/2 complexes, hydrolyzes Rheb-GTP, and inhibits mTORC1 activity, thereby decreasing catabolism and increasing anabolism. **b** Schematic showing REDD1-mediated atypical NF-κB activation. NF-κB activation is generally triggered either by the IKKαβ-dependent canonical pathway or the IKKα-mediated noncanonical pathway following the ligation of cytokine or Toll-like receptors (R) via their cognate ligands (L). Furthermore, REDD1 activates the atypical NF-κB pathway by interacting with and sequestering IκBα, liberating NF-κB p65/50 from IκBα, and translocating it to the nucleus.

The serine/threonine protein kinase mTOR exists in two distinct complexes, mTORC1 and mTORC2, and functions as a central signaling hub that integrates networks involved in cellular metabolism, energy homeostasis, and cell growth^78^. mTORC1 not only promotes the biosynthesis of proteins, lipids, and nucleotides required for cell growth and proliferation but also inhibits catabolism, which degrades intracellular bulk proteins and damaged organelles, by repressing lysosome biogenesis and autophagy (Fig. 2a).

mTORC1 activity is controlled by two upstream negative regulators, REDD1 and a complex comprising tuberous sclerosis protein 1 (TSC1) and TSC2^15,79^. In general, mTOR activity is repressed by TSC1/2 under physiological conditions and promoted by TSC1/2 inactivation following stimulation with growth factors^15^. When exposed to hypoxia- or energy-induced stress, which stimulates REDD1 expression, mTORC1 activity was inhibited in wild-type (WT) cells but not in *Tsc2*-deficient cells^15,80^, suggesting that REDD1 inhibits mTORC1 by maintaining TSC1/2 activity. TSC1 predominantly localizes to endomembranes and stabilizes TSC2 by forming a complex with it^81,82^, whereas TSC2 functions as a GTPase-activating protein (GAP) toward the small GTPase Rheb^83^. Human TSC2 can be phosphorylated at multiple amino acid residues by several upstream kinases, including Akt, ERK, AMPK, GSK3β, and IKKα^84^. Among the TSC2 amino acids phosphorylated by growth factor-activated Akt^85,86^, Ser939 and Ser981 are the most important for TSC2-mediated mTORC1 inhibition^81,86,87^. Phosphorylation at both Ser939 and Ser981 does not alter intrinsic TSC2 GAP activity toward Rheb but contributes to the translocation of TSC2 from endomembranes to the cytosol through binding to 14-3-3 proteins, resulting in loss of its GAP activity and subsequent hyperactivation mTORC1^15,81^ (Fig. 2a).

As briefly described above, REDD1 inhibits mTORC1 signaling by maintaining TSC2 GAP activity. However, owing to its lack of catalytic activity^15,74,77^, REDD1 binds to 14-3-3 proteins and dissociates the inactive TSC2/14-3-3 complex to maintain GAP activity and subsequently inhibit mTORC1 activation (Fig. 2a). REDD1 colocalizes with TSC2 in the endomembrane system, increases its local concentration relative to 14-3-3 proteins, and increases its ability to sequester 14-3-3 proteins, thus preventing TSC2/14-3-3 association^15^. The REDD1 sequence ^133^RLAYSEP^139^ may interact with 14-3-3 proteins^74^, because it is similar to the canonical 14-3-3 binding motif RXXX(pS/T)XP^88^. Furthermore, REDD1 Arg133 and Pro139 are essential for the interaction with 14-3-3 proteins and inhibition of mTORC1 activity^15^, but it is still unclear whether the ^133^RLAYSEP^139^ sequence binds directly to 14-3-3 proteins.

REDD1 can also inhibit mTOR activity through its ability to promote protein phosphatase 2A (PP2A)-mediated inactivation of Akt, which is an upstream regulator of TSC2^89^. REDD1 binds to PP2A and Akt and inhibits Akt activity through PP2A-mediated dephosphorylation of Thr308 but not Ser473, although both residues are essential for the full activation of the kinase. Inactivated Akt reduces TSC2 phosphorylation at Ser939 and Ser981 and blocks the formation of inactive TSC2/14-3-3 complexes, thus preventing mTORC1 activation. Furthermore, REDD1 induced by amino acid deprivation reduces Akt phosphorylation at Ser473, although Ser308 phosphorylation was not measured^33^. However, hypoxia-induced REDD1 blocks TSC2/14-3-3 association without altering Akt or TSC2 phosphorylation^15^. Therefore, it is unclear whether REDD1 inhibits mTORC1 via the PP2A−Akt axis. Although REDD1 inhibits mTORC1 activity through dual functions as a 14-3-3 sequestrant and Akt inactivator, the underlying molecular mechanisms are likely more complex than originally anticipated.

### REDD1 as an activator of the atypical NF-κB pathway

The transcription factor NF-κB regulates the expression of many genes involved in a variety of biological processes, including the immune response, cell growth and survival, and development. Activation of NF-κB is mediated mainly by two major signaling pathways, the canonical and noncanonical pathways (Fig. 2b), which are activated by the TAK1/IKKαβ- and NIK/IKKα-dependent pathways, respectively^90^. Furthermore, another NF-κB activation pathway, called the atypical NF-κB pathway, is activated through an IKK-independent mechanism by noninfectious environmental stressors^91–93^.

REDD1 is likely involved in NF-κB activation and inflammation under various pathological stress conditions^6,14,61,62^. Cigarette smoke-induced REDD1 or exogenously overexpressed REDD1 promoted NF-κB-dependent inflammation in cultured mouse bronchoalveolar lavage cells and lung fibroblasts as well as in mouse models^14^. Furthermore, LPS increased REDD1 expression in immune cells and stimulated higher NF-κB activation, cytokine gene expression, and inflammasome activation in WT cells than in *Redd1*-knockdown or *Redd1*-deficient cells^60–62,94^, further contributing to the prolonged or delayed inflammatory response of the innate immune system^61^. Therefore, stress-induced REDD1 appears to be an important regulatory factor in NF-κB-dependent inflammation.

As an endogenous mTORC1 inhibitor, REDD1 may stimulate NF-κB activation by inhibiting the mTORC1 signaling pathway. LPS-induced NF-κB activation and cytokine production were reduced in *Redd1*- and *Tsc2*-deficient immune cells, and these effects were restored by rapamycin^94,95^, indicating that REDD1 stimulates NF-κB activation and the inflammatory response by inhibiting mTORC1 activation. Similarly, three independent groups reported that NF-κB activation and inflammatory responses induced by cigarette smoke or LPS were decreased in *Redd1*-deficient mice or macrophages; however, these effects were not or only partially restored by treatment with the mTORC1 inhibitor Torin2 or rapamycin^13,61,62^. Therefore, despite being an mTORC1 inhibitor, REDD1 activates NF-κB largely independent of mTORC1 action.

REDD1 plays an important role in the pathogenesis of metabolic disorders, which are closely associated with low-grade inflammation, also known as meta-inflammation^6,96^, suggesting that REDD1 contributes to metabolic disorders via its promotion of NF-κB-dependent systemic inflammation. Given that obesity-induced chronic low-grade inflammation differs to some degree from innate and adaptive immunity^97^, REDD1 may trigger atypical NF-κB activation independent of the IKK-dependent canonical and noncanonical pathways^6,61^. The atypical NF-κB pathway can be activated by physiological, oxidative, and genotoxic stressors^92,93,98^, all of which can induce REDD1. REDD1 overexpression activated NF-κB and inflammation without triggering the IKK-dependent canonical and noncanonical pathways that depend on the phosphorylation or degradation of TAK1, NIK, IKKα/β, IκBα, and p100^61^, although it has been reported to promote IKK-mediated canonical NF-κB signaling^28^. Moreover, REDD1-induced NF-κB activation was ablated by knockdown of NF-κB *p65* but not of IKKα and IKKβ^6^. These results suggest that REDD1 activates NF-κB via the atypical pathway.

**Fig. 3:**
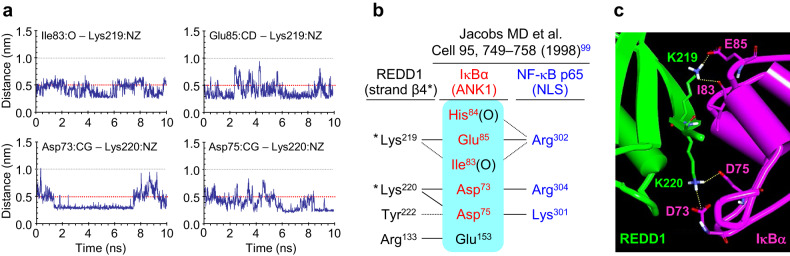
Molecular interaction between the top-scoring hit amino acids of REDD1 and IκBα. **a** Trajectories (10 ns) of Ile83:O–Lys219:NZ, Glu85:CD–Lys219:NZ, Asp73:CG–Lys220:NZ, and Asp75:CG–Lys220:NZ. **b** Details of the interactions between amino acids in IκBα (residues 67−103 in ankyrin repeat domain 1, ANK1) and REDD1 (residues 214–220 in strand β4, indicated with *) or NF-κB p65 (nuclear localization sequence, NLS, ^301^KRKR^304^). The solid and dotted lines indicate salt bridges and hydrogen bonds, respectively. **c** Molecular interaction model showing of REED1 and IκBα. Lys219 and Lys220 in strand β4 of REDD1 are likely to form hydrogen bonds and salt bridges with Asp73, Asp75, Ile83, and Glu85 in ANK1 of IκBα.

REDD1 can physically interact with IκBα, either liberating NF-κB from inactive IκBα/NF-κB complexes or preventing the formation of these complexes, thus increasing NF-κB nuclear translocation and inflammatory gene expression (Fig. 2b). The REDD1 C-terminal region (amino acid residues 178–229) plays a crucial role in NF-κB activation through physical interaction with and sequestration of IαBα in the cytoplasm^61^. Among the amino acid residues in the C-terminal region, Lys219 and Lys220 have been shown to form hydrogen bonds and salt bridges with the ankyrin repeat domain 1 (ANK1, amino acid residues 67–103) of IκBα^6^ (Fig. 3a-c). These interactions block IκBα binding to NF-κB p65 by hindering the approach of Asp73, Asp75, Ile83, and Glu85 of ANK1 to the p65 nuclear localization signal (^301^KRKR^304^), allowing NF-κB to be liberated from its inactive complex and translocated to the nucleus^6^^,99^. This mechanism was confirmed by the significant reduction in NF-κB-mediated inflammation in cells overexpressing mutant *Redd1*^K219A/K220A^ and by the suppression of proinflammatory cytokine production in high-fat diet (HFD)-fed *Redd1*^K219A/K220A^ mutant mice^6^. Therefore, REDD1 seems to stimulate meta-inflammation through atypical activation of NF-κB activation by sequestering IκBα. Notably, REDD1 preferentially interacts with newly synthesized free IκBα rather than with IκBα bound to the NF-κB p65/p50 dimer, inducing a delayed or sustained inflammatory response after LPS stimulation^61^ or stimulating low-grade inflammation under obesity-induced energy stress conditions^6^ (Fig. 2b).

### REDD1 as a pro-oxidant or antioxidant

REDD1 exhibits dual but opposite functions against oxidative stress, such as increased intracellular ROS accumulation^2^ or reduced mitochondrial ROS production^17^, depending on the type of cells and stressors and its cellular location. REDD1 shows pro-oxidant activity, as indicated by increased and decreased intracellular ROS levels in REDD1-overexpressing cells and *TP63*-null fibroblasts (which do not express REDD1), respectively^20^. In addition, *TP63*-null cells prevented exogenous H_2_O_2_-mediated elevation of intracellular ROS levels, in contrast to the effect of REDD1- or TP63-reconstituted *TP63*-null cells. Moreover, hyperglycemia increased REDD1 expression and ROS production in R28 retinal cells, and these effects were attenuated by *Redd1* knockdown^71^. These results indicate that REDD1 functions as a pro-oxidant that inhibits cellular redox activity. Exposure to hypoxia, inducing REDD1 expression, increases cellular ROS levels and autophagic flux in primary fibroblasts by decreasing the cellular redox potential, but this mechanism is not triggered in *Redd1*- or *Tsc2*-deficient cells^2^. Moreover, defective autophagy in *Redd1*-deficient cells was only partially restored by rapamycin, and overexpression of a *Redd1*-RPAA mutant that fails to activate mTORC1 was sufficient to induce ROS-mediated autophagy^2^. These findings indicate that REDD1 elevates intracellular ROS levels and autophagy independently of mTORC1 activity.

Since mTORC1 blocks autophagy through inhibitory phosphorylation of ULK1 at Ser757, the question remains, how does REDD1 stimulate mTORC1-independent autophagy? REDD1-induced ROS production, as shown previously^17,20^, promotes autophagy by inhibiting the delipidating activity of ATG4B on lipid-conjugated LC3 through the formation of a disulfide bond between redox-sensitive Cys292 and Cys361 residues^2,100–102^. Another question is, how does REDD1 induce ROS production? Hypoxia or starvation leads to increased cytosolic ROS levels and subsequently stimulated autophagy through coexpression of REDD1 and the pro-oxidant thioredoxin-interacting protein (TXNIP), which form a complex; however, all these effects were attenuated in *Redd1*-deficient cells^2^. Moreover, TXNIP overexpression or REDD1 reconstitution in *Redd1*-deficient cells did not affect or led to modest increases in intracellular ROS levels, while coexpression of both genes synergistically increased cytosolic ROS levels by inhibiting antioxidant thioredoxin (Trx) activity^2^. This finding indicates that REDD1 elevates cellular ROS levels by cross-interacting with TXNIP and subsequently activates oxidative stress-induced autophagy. Similarly, HeLa cells cultured with 2-deoxyglucose (an inhibitor of glycolysis) and mouse hearts undergoing ischemia/reperfusion coexpressed REDD1 and TXNIP and promoted REDD1-dependent mTOR inhibition and TXNIP-mediated autophagy, which were attenuated by *Txnip* deletion or *Redd1* knockdown^25,103^, further supporting the idea that REDD1 and TXNIP synergistically enhance their own biological activities.

**Fig. 4:**
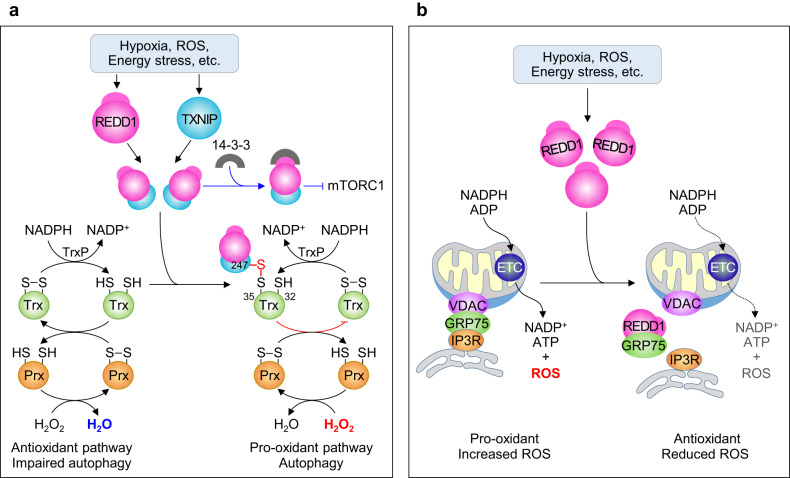
Models of REDD1-mediated pro-oxidant or antioxidant activity. **a** A schematic model of REDD1 as a pro-oxidant. REDD1 and TXNIP are unstable but are stabilized by forming a disulfide bond-mediated dimeric complex between Cys247 of TXINP and Cys32 of thioredoxin (Trx), thereby resulting in the inhibition of the Trx-thioredoxin reductase (TrxR) system coupled with the redox cycle involving peroxiredoxin (Prx), accumulation of cytosolic ROS, and promotion of oxidative stress and autophagy. **b** A schematic model of REDD1 as an antioxidant. Mitochondria-associated endoplasmic reticulum membranes (MAMs) are stabilized by the protein complexes composed of voltage-dependent anion channel (VDAC), 75 kDa glucose-regulated protein (GRP75), and inositol 1,4,5-trisphosphate receptor (IP3R). MAMs promote mitochondrial function, particularly electron transport chain (ETC) activity coupled with ROS production, leading to mitochondrial APT and ROS production. However, REDD1 disrupts MAM structure by interacting with and sequestering GRP75, resulting in a decrease in mitochondrial ROS production.

As both REDD1 and TXNIP are unstable proteins with half-lives of ~10 min^75,104,105^, their biological activities can be controlled by changes in their stability, which is regulated by protein–protein interactions, posttranslational modifications, and the ubiquitin–proteasome pathway^106^. Both proteins are rapidly degraded by the ubiquitin‒proteasome pathways associated with the CUL4A−DDB1−ROC1−β-TRCP E3 ligase complex and the E3 ligase ITCH, respectively^77,107^, but the interactions between REDD1 and TXNIP reduce their affinity for the E3 ligase systems and thus lead to their resistance to proteasomal degradation. Physical interactions between both proteins were initially identified using a yeast two-hybrid screening system and were confirmed by coimmunoprecipitation assay^25^. REDD1 interacts with the C-terminal region (1–155 amino acid residues) of TXNIP, indicating that the REDD1/TXNIP complex may increase their own stability and activity, thus promoting REDD1-dependent mTORC1 inhibition^25,103^ and TXNIP-mediated cellular ROS production and accumulation^7^ (Fig. 4a). Generally, TXNIP inhibits the Trx-Trx reductase (TrxR)−NADPH axis by forming a mixed disulfide bond between TXNIP Cys247 and redox-active Trx Cys32, leading to reduced antioxidant activity in cells^108^. These findings indicate that the physical interactions between REDD1 and TXNIP mutually enhance the stability and activity of each protein, thereby inhibiting mTORC1 activation and the antioxidant Trx activity (Fig. 4a).

In contrast, however, REDD1 elicits antioxidant activity, as indicated by lower mitochondrial ROS production in WT cells than in *Redd1*-deficient cells as well as in *Redd1*-overexpressing cells^17,19^. Notably, REDD1 localizes to mitochondria, where it is required for inhibiting mitochondrial ROS generation^17^, indicating that REDD1 exerts an indirect antioxidant effect. Notably, REDD1 disrupts the structure of mitochondria-associated ER membranes (MAMs) by directly binding to 75 kDa glucose-regulated protein (GRP75), which is a crucial component for the formation and stabilization of complexes with both the inositol 1,4,5-trisphosphate receptor on the ER and voltage-dependent anion channels on the mitochondrial outer membrane at sites of close contact between the ER and mitochondria^5^. REDD1-induced disruption of MAM integrity decreases the mitochondrial metabolic rate, resulting in reduced O_2_ consumption and ATP synthesis^5^, because MAMs promote mitochondrial respiration and bioenergetics by facilitating Ca^2+^ transport from the ER to mitochondria^109^. As a result, REDD1 decreases the efficiency of the mitochondrial electron transport chain, which is coupled with ROS production^110^, thereby reducing mitochondrial ROS production (Fig. 4b). Collectively, these results suggest that REDD1 functions as a pro-oxidant or antioxidant by either reducing cytosolic redox activity or decreasing mitochondrial ROS production, presumably depending on its interaction partner (TXNIP vs. GRP75) or cellular localization (MAMs vs. cytosol).

## Pathophysiological roles of REDD1

**Fig. 5:**
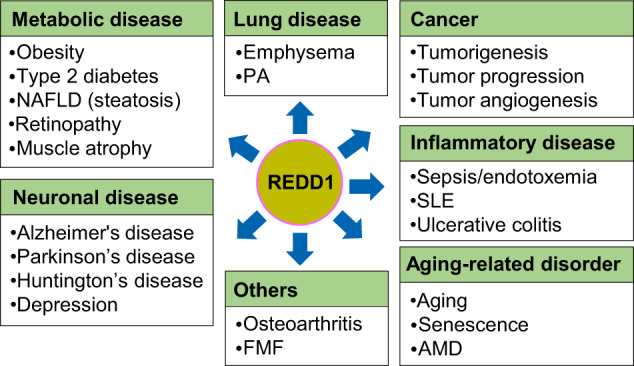
Various types of diseases associated with REDD1. AMD age-related macular degeneration, FMF familial Mediterranean fever, NAFLD nonalcoholic fatty liver disease, PH pulmonary hypertension, SLE systemic lupus erythematosus.

REDD1 expression increases in vitro and in vivo under various pathophysiological stress conditions, including metabolic imbalance, hypoxia, inflammation, stress hormones, and aging. Therefore, REDD1 is likely an important risk factor for the development of various diseases, which are listed in Fig. 5.

### REDD1 in obesity

Mice with global knockout of S6K1 or adipocyte-specific deletion of Raptor (a downstream effector or regulatory subunit of mTORC1, respectively) exhibited protection against obesity^111,112^, suggesting the possibility that REDD1-mediated inhibition of the mTORC1 pathway prevents the development of obesity. REDD1 was found to be upregulated in *ob*/*ob* and HFD-induced obese mice; however, unexpectedly, mTORC1 was highly activated in both models^9^. When fed an HFD, *Redd1*-deficient mice gained less body weight than WT mice, but mTORC1 signaling was maintained at a high level in both groups of mice, indicating that REDD1 promotes obesity independently of mTORC1. Furthermore, REDD1 expression was increased in the liver tissues of HFD-induced obese mice and patients with morbid obesity, and *Redd1* deletion protected mice from HFD-induced increases in liver weight and hepatic steatosis; however, unexpectedly, it also reduced mTORC1 signaling^7^. Thus, the anti-steatosis effect of *Redd1* deficiency was associated with decreased lipogenic gene expression, probably by decreasing the lipid anabolic mTORC1 pathway^113^. Interestingly, REDD1 has been shown to regulate adipogenesis in a tissue-specific manner, as shown by the significant deduction of fat deposition in gonadal white adipose tissue and the expansion of dermal white adipose tissue in *Redd1*-deficient mice fed an HFD^114^. Moreover, deletion of *Redd1* in the whole body or only in adipocytes protected mice against HFD-induced adipogenesis, obesity, and steatosis without affecting the mTORC1 pathway^6^. *Redd1* deficiency also increased the basal metabolic rate, probably by promoting MAM integrity and mitochondrial metabolism, and reduced weight gain and white adipose tissue deposits in aged mice^5^. These results suggest that REDD1 is an important contributor to obesity development, because it increases adipogenesis and reduces the metabolic rate.

Several recent studies have demonstrated the molecular mechanisms through which REDD1 promotes adipogenic differentiation and adipogenesis. REDD1 is upregulated during preadipocyte differentiation, which is abrogated by *Redd1* deletion or knockdown^6^. Adipogenic differentiation is stimulated in *Redd1*-overexpressing preadipocytes but not in cells transfected with *Redd1*^K219A/K220A^, which does not inhibit atypical NF-κB activation^6^. NF-κB inhibition prevents obesity development by downregulating the adipogenic transcription factors CEBPα and PPARγ^115^. NF-κB activation stimulates CEBPα expression by binding to κB sites in its promoter, subsequently promoting PPARγ expression via a positive cross-regulation loop and in turn increasing lipogenic gene expression^6,116^. Therefore, the anti-obesity effect observed in *Redd1*-deficient and *Redd1*^K219A/K220A^ mutant mice is likely due to the suppression of adipogenic differentiation, lipogenesis, and adipocyte hypertrophy independently of mTORC1 signaling^6^. Collectively, REDD1 is sufficient to drive weight gain and ultimately obesity via atypical NF-κB activation rather than via mTORC1 pathway regulation.

### REDD1 in skeletal muscle atrophy

Skeletal muscle is a plastic organ maintained by multiple pathways that regulate the balance between protein synthesis and degradation. Glucocorticoids cause muscle atrophy by impairing protein synthesis and promoting protein degradation. As REDD1 is a glucocorticoid-responsive protein and acts as an inhibitor of mTORC1 that promoting anabolic and inhibiting catabolic pathways through the phosphorylation of target substrates^3^, this protein is involved in skeletal muscle atrophy. Infusion of the synthetic glucocorticoid DEX into rats resulted in increased REDD1 levels, impaired mTORC1 activation, and caused muscle atrophy in WT mice but not in *Redd1*-deficient mice^4,47^. Fasting or starvation caused a marked increase in endogenous corticosterone levels and REDD1 expression and significantly reduced mTORC1-mediated protein synthesis in the skeletal muscle of mice, which was restored in *Redd1*-deficient mice^33,117,118^. Furthermore, treatment with doxorubicin or carboplatin resulted in REDD1-mediated mTORC1 inhibition and muscle atrophy, resulting in decreased running capacity in mice^18,119^, and these outcomes were prevented in *Redd1*-deficient mice or by administration of branched-chain amino acids that are potent activator of mTORC1^4,120,121^. These results indicate that REDD1 is sufficient to induce muscle atrophy by inhibiting mTORC1-dependent protein synthesis under pathological stress conditions.

In addition, REDD1 triggers proteolysis and causes the removal of damaged organelles by activating autophagy through the negative regulation of the TXNIP–ROS–ATG4B axis^2^ and/or, at least in part, the mTORC1–ULK1 axis^3,122^. DEX treatment resulted in REDD1 induction, mTORC1 inhibition, protein synthesis inhibition, and muscle atrophy, along with increased ULK1 activation and autophagy in mice, all of which were prevented in *Redd1*-deficient mice^4^. Similar results were observed in cultured rat skeletal muscle cells^123^. These results indicate that REDD1 induces muscle atrophy via protein synthesis inhibition and autophagy induction, both of which are driven by REDD1-dependent mTORC1 inhibition. Moreover, the REDD1–TXNIP–ROS–ATG4 autophagy axis may also be involved in muscle- wasting syndromes^2^.

Aging is accompanied by sarcopenia, a type of muscle atrophy characterized by progressive loss of skeletal muscle mass and function, resulting in physical disability. As mTORC1 drives most anabolic processes, REDD1-mediated mTORC1 inhibition may also be important for age-related muscle atrophy. Physical exercise results in higher REDD1 expression and lower mTORC1 activation and protein synthesis in muscle biopsy specimens from older people than in those from younger individuals^124,125^, and aged gerbils presented markedly increased REDD1 expression and decreased mTORC1 activation^58^. Therefore, age-related REDD1 upregulation may contribute to sarcopenia by inhibiting mTORC1-mediated protein synthesis.

### REDD1 in diabetes and retinopathy

Obesity stimulates meta-inflammation, which contributes to obesity-linked metabolic disorders, such as insulin resistance and T2D^6^. Peripheral glucose uptake is significantly increased in response to insulin in obese rats administered a recombinant antagonist against TNF-α^126^, suggesting that NF-κB-mediated inflammation contributes to the development of insulin resistance and obesity-associated diabetes. Genetic or pharmacological inhibition of the NF-κB pathway restores insulin sensitivity and weight gain in HFD-induced obese mice^127–129^. Notably, palmitate, a REDD1 inducer and insulin resistance factor, increases NF-κB-mediated cytokine production^31,129^. Moreover, REDD1 stimulates IKK-independent atypical NF-κB activation and TNF-α and IL-1β production in obese mice^6^.

TNF-α and IL-1β induce the inhibitory phosphorylation of multiple serine/threonine residues on insulin receptor substrate-1 (IRS-1) and IRS-2 by activating JNK and IKKβ, subsequently inhibiting the PI3K-Akt pathway^130,131^. REDD1 expression was increased in cultured cells exposed to diabetic conditions and in skeletal muscle in diabetic mice and patients^26,38,71,132^ and hindered glucose uptake and glycolysis^8^. REDD1 also plays an important role in insulin signaling, as evidenced by significantly lower insulin-induced Akt phosphorylation levels in adipocytes cocultured with LPS-stimulated WT macrophages than in those cocultured with *Redd1*-deficient macrophages due to the crucial involvement of REDD1 in NF-κB-mediated cytokine production^62^. Overall, REDD1 stimulates atypical NF-κB activation-mediated proinflammatory cytokine production, thereby contributing to T2D through inhibition of an insulin-driven IRS−PI3K−Akt signaling axis^6^.

Retinopathy, one of the major complications of diabetes, is characterized by functional impairment of the retinal microvasculature, leading to hemorrhaging, angiogenesis, retinal detachment, and blindness. REDD1 has been implicated in the pathogenesis of diabetic retinopathy^10,11^. The pathophysiological role of REDD1 in retinopathy was first demonstrated in a model of retinopathy of prematurity using *Redd1*-deficient mice^11^, as evidenced by a reduction in retinal neovascularization and apoptosis without altering retinal levels of vascular endothelial growth factor (VEGF), which is a major risk factor for retinopathy^133^, compared with WT mice. Treatment with streptozotocin, a diabetes inducer, resulted in a significant increase in retinal apoptosis and neuroretinal dysfunction in WT mice but not in *Redd1*-deficient mice^36^, supporting the idea that REDD1 plays a role in diabetes-induced retinal neurodegeneration. The pathogenic functions of REDD1 in diabetic visual dysfunction are considered to involve ROS generation and oxidative stress^13^ by increasing TXNIP-mediated pro-oxidant activity independently of mTORC1 inhibition^2,71^. REDD1-mediated NF-κB activation may also play an important role in the pathogenesis of retinopathy, as *Redd1* deletion prevented NF-κB activation and subsequently improved visual acuity in diabetic mice^28^. Overall, REDD1 is an important causative factor of diabetic retinopathy, because it stimulates ROS production or NF-κB-dependent inflammation in the retina. Therefore, a siRNA targeting *Redd1* may be useful in therapeutic strategies for diabetic macular edema or age-related macular degeneration^134^^,^^135^.

### REDD1 in neurological disorders

The mTORC1 pathway is activated in various neurological diseases, including tuberous sclerosis, neurodegeneration, and autism^136^, and can be used as a therapeutic target for these diseases^137^^,138^, suggesting that REDD1 expression or activity may be reduced in neurodegenerative diseases. However, unexpectedly, REED1 is upregulated and inhibits mTOR signaling in the hippocampus of aged rats^58^ and in the brain tissues of patients with neurodegenerative diseases, including Alzheimer′s disease (AD) and Parkinson’s disease (PD)^139^. These findings suggest that REDD1 induction may be relevant in the pathogenesis of neurodegeneration and related diseases.

REDD1 upregulation in the central nervous system might exert a detrimental effect on neuronal activity and function through inhibition of mTOR-dependent protein synthesis. Exposure to chronic unpredictable stress increased REDD1 expression in the rat prefrontal cortex, as also observed in patients with major depressive disorder, and concomitantly decreased mTORC1 activation and protein synthesis-dependent synaptogenesis, resulting in synaptic loss, neuronal atrophy, and depression- and anxiety-like behaviors^12^. Similar effects have also been observed in *Redd1*-overexpressing mice but not in *Redd1*-deficient mice. These results suggest that REDD1 is sufficient to induce neuronal atrophy by inhibiting mTORC1-dependent protein synthesis. However, the neurodevelopmental disorder tuberous sclerosis is caused by mutations in the TSC1/2 complex, known as an mTORC1 inhibitor and a REDD1 effector, and mTORC1 signaling was hyperactivated in the brains of most of these patients^139^^,^^140^. Moreover, these patients exhibit a high incidence of autistic behaviors and depressive and anxiety disorders^140^, indicating that *Tsc1*/*2* mutations trigger hyperactivation of the mTOR signaling pathway, causing abnormal cell growth/proliferation that lead to developmental neurological disorders. Therefore, homeostatic control of the REDD1−TSC1/2−mTORC1 signaling axis is required for normal neurodevelopment and prevention of neuronal disorders.

Furthermore, REDD1 may be involved in the pathogenic development of neurodegenerative diseases associated with memory and cognitive impairment^141^^,^^142^. REDD1 was upregulated in human neuroblastoma cells treated with Aβ, and Aβ-induced cytotoxicity was increased after REDD1 was overexpressed, but this effect was prevented when REDD1 was knocked down^53^, implicating REDD1 in AD pathogenesis. Aβ treatment increased REDD1 expression in mouse hippocampal slices, inhibited mTOR signaling, and blocked synaptic plasticity, all of which were suppressed after *Redd1* knockdown. Moreover, direct injection of Aβ into the lateral ventricle of mice impaired recognition memory, which was blocked by *Redd1* knockdown^141^. The REDD1 level was increased in the hippocampus of 5XFAD mice (an AD mouse model) and lymphocytes of patients with AD, and local *Redd1* knockdown ameliorated cognitive deficits in 5XFAD mice^143^^,^^144^. Therefore, REDD1 is involved in Aβ-induced synaptic dysfunction and memory impairment in an AD-like mouse model.

In addition, the REDD1 level was increased in the substantia nigra neurons of patients with PD, and treatment with the dopaminergic neurotoxin 6-OHDA increased REDD1 expression and cell death, along with inhibition mTORC1 activity in neuronal PC12 cells^145^^,^^146^. Exposure to chronic restraint stress accelerated the pathological process leading to PD-like symptoms in a PD-sensitive animal model, and behavioral defects were improved by *Redd1* knockdown^142^. Moreover, pharmacological or genetic inhibition of TXNIP activity prevented detrimental outcomes of neurodegenerative diseases^147^. Therefore, both REDD1 and TXNIP may be involved in the pathogenesis of neurological disorders and neurodegenerative diseases by inhibiting protein synthesis and ROS-mediated oxidative stress^2^; however, the REDD1−NF-κB pathway may also play a role, as inflammation is a contributing factor to neurodegenerative diseases^148^.

### REDD1 in cancers

mTORC1 promotes anabolic metabolism and tumor progression, and rapamycin analogs inhibit the growth of several tumor-derived cell lines in vitro and in vivo^149^. In this respect, REDD1 shows antitumor activity and is likely downregulated in tumors. REDD1 expression is reduced in human breast and pancreatic cancer specimens compared to that in patient-matched normal tissues^15^, indicating that REDD1 suppresses tumor growth and metastasis. REDD1 inhibits cancer initiation and progression, as evidenced by an increase in tumorigenesis, tumor growth, and metastasis of immortalized *Redd1*-deficient cells or *Redd1*-knockdown *Kras*^*G12D/+*^ pancreatic neoplasms in mouse models^15,17,150^. The tumorigenicity of *Redd1*-deficient cells is dependent on mTORC1 activation and mitochondrial ROS production^15,17^. Human hepatocellular carcinomas (HCCs) with inactive *Tsc2* mutations exhibit more aggressive tumor behavior in patients, and *Tsc2* mutation-bearing HCCs are more sensitive to rapamycin in patient-derived tumor xenograft models^151^. These findings suggest that REDD1 downregulation promotes tumor progression by stimulating mTORC1-mediated tumor cell proliferation^15^ or by increasing the levels of mitochondrial ROS as a regulator of HIF-1-dependent tumorigenic metabolism^17^. Therefore, upregulation of REDD1 by treatment with various chemotherapeutic drugs has been associated with decreased viability of breast cancer cells^46,49^. In contrast, REDD1 is upregulated in various types of cancers, such as myeloid leukemia, glioblastomas, carcinomas, gastric cancers, and breast cancers, resulting in poor prognosis, aggressive malignancy, and reduced overall and disease-free survival in cancer patients^152–155^. Furthermore, a meta-analysis showed that high levels of REDD1 were associated with a worse prognosis in acute myeloid leukemia, breast cancer, glioblastoma, and colon and lung cancer but, in contrast, better prognosis in gastric cancer^156^. These results suggest that REDD1 exhibits either oncogenic or tumor-suppressive functions, depending on the cell type and cellular context.

*Redd1* deficiency reprograms lipid metabolism to drive the invasion and metastasis of *Ras*-mutant tumors in mice. Furthermore, decreased REDD1 levels can predict poor patient survival specifically in *Ras*-mutant lung and pancreatic carcinomas^150^. The tumor microenvironment is composed of various nonmalignant cell types, including tumor-associated macrophages (TAMs) and endothelial cells (TECs), which play important roles in tumor progression^157^. *Redd1*-deficient TAMs enhances glucose uptake and glycolysis via mTORC1-dependent GLUT1 upregulation, resulting in low glucose availability and quiescence in TECs; quiescent TECs maintain vascular integrity, thus inhibiting metastasis^8^. Treatment with low-dose doxorubicin or cisplatin elevates REDD1 expression and reduces mTORC1-dependent translation of VEGF receptor-2/3 and eNOS in TECs and endothelial progenitor cells, all of these effects are inhibited in *Redd1*-deficient cells, resulting in suppressed tumor angiogenesis and lymphangiogenesis, thereby inhibiting tumor growth and metastasis^18,158^. Overall, although some results are debated, REDD1 shows cell type-specific functions in inhibiting tumorigenesis, tumor progression, and metastasis.

### REDD1 in inflammatory diseases

NF-κB is a central mediator of inflammation and its related diseases by stimulating proinflammatory cytokine production. REDD1 levels correlated with NF-κB activation and inflammation in patients and mice with obstructive airway diseases, and *Redd1* deficiency prevented NF-κB activation, alveolar inflammation, and emphysema-like symptoms in mice chronically exposed to cigarette smoke^14^. LPS increased REDD1 expression, which in turn stimulated atypical NF-κB activation and inflammatory cytokine production, and these effects were attenuated by *Redd1* deletion or knockdown^60–62^^,94^. As expected, *Redd1* downregulation protected against inflammatory diseases, including endotoxemia and endothelial cell injury^61,94,159^. Considering these outcomes, REDD1 is a useful target for the treatment of endotoxemia and related inflammatory diseases.

Furthermore, REDD1 was upregulated in primary human T cells or mouse splenocytes stimulated with phytohemagglutinin (PHA), and PHA-induced proliferation of mouse CD4 T cells was diminished by *Redd1* deletion^160^. *Redd1*-deficient T cells were more likely to die than WT cells. Therefore, REDD1 may contribute to the development of autoimmune diseases by promoting T-cell proliferation. DEX activates autophagy in mouse thymocytes and murine lymphoma cells through REDD1 induction^3^, and autophagy is frequently activated in T cells and B cells from a lupus mouse model and in blood mononuclear cells from patients with systemic lupus erythematosus (SLE)^161^, indicating that REDD1-mediated autophagy is associated with SLE. Indeed, SLE patient-derived blood neutrophils show increased REDD1 expression and basal autophagy, along with the formation of enhanced neutrophil extracellular traps (NETs), through a process known as NETosis, resulting in skin inflammation and renal fibrosis through the upregulation of tissue factor and IL-17A^162^. REDD1 is also critical for autophagy and chronic intestinal inflammation in patients with active ulcerative colitis^163^. These effects suggest that REDD1 stimulates T-cell proliferation, autophagy, and NETosis to induce inflammation and organ damage during autoimmune disease development.

Considering its chronic inflammatory function^6,14,61^, REDD1 may be involved in the pathogenesis of rheumatoid arthritis, known as an autoimmune disorder. However, its potential pathogenic role has not yet been studied. Osteoarthritis (OA) is a degenerative joint disease, and high levels of inflammatory cytokines have been found in the synovium of patients with early-stage OA^164^. REDD1 may contribute to the pathogenesis of OA via the REDD1−NF-κB−inflammation axis. However, unexpectedly, REDD1 levels were found to be reduced in the cartilage of patients with OA, and *Redd1* deficiency exacerbated the acquisition of OA phenotypes in an mTORC1-dependent manner in cultured chondrocytes and experimental OA mouse models^1,165^. Future studies should explore and identify the role of the REDD1−NF-κB axis in rheumatoid arthritis and OA.

## Conclusions and future perspectives

Stress-responsive REDD1 plays an important role in cellular homeostasis and the pathogenesis of various diseases. REDD1 is induced by DNA damage, hypoxia, glucocorticoids, metabolic stress, and several other cellular stressors. REDD1 regulates cellular function and activity in an mTORC1-dependent or -independent manner. This protein regulates cellular metabolism, low-grade inflammation, and cellular redox potential through the following three mechanisms: mTORC1 inhibition by blocking the TSC2/14-3-3 association, atypical NF-κB activation by sequestering IκBα, and enhanced cellular pro-oxidant or antioxidant activity by stabilizing the TXNIP protein or disrupting MAM integrity. Generally, REDD1 functions as a mediator in the development or progression of many diseases, including metabolic disorders, cancer, muscle atrophy, neurological diseases, and autoimmune diseases; however, in some cases, the results are debatable. As the dual effects of REDD1 on disease pathogenesis may depend on its interaction partners or subcellular localization, cell type, and cellular context, further studies performed under clearly defined and controlled conditions and factors are needed. Additionally, given the emerging evidence for REDD1 involvement in the development of several diseases, further research should be conducted to develop therapeutic strategies, including chemical and biological drugs, to target REDD1 based on its underlying molecular mechanism.
